# Prescription Department

**Published:** 1891-08

**Authors:** 


COUGH MIXTURE.

The following is said by the *College and Clinical Record* to be Dr. E. G. Janeway's favorite prescription:

℞ Syrup tolu.
Syrup pruni virginian.
Tinct. hyoscyami.
Spirit ætheris comp.
Aquæ, aa oz. j.
M. Sig. Dose, a teaspoonful.

TREATMENT OF BALANITIS.

Dr. W. R. Chichester states that he has obtained good results from the employment of the following:

℞ Atropiæ sulphatis, gr. j.
Zinci sulphatis, grs. ij.
Acid boracic, grs. v.
Aquæ distillat, oz. j.

M. Sig. Apply two or three times daily with a brush.

He further states that this is open to any modification which the case suggests.

CHRONIC GONORRHOEA.

Where the stomach will not tolerate Bumstead's copaiba, iron and cantharidis, or similar mixtures in gleety cases of gonorrhœa, use—

℞ Elixir lactopeptine, gent. and chlo. iron, oz. iss.
Tr. cantharidis, dr. ij.
Spir. juniper, dr. vj.

Sig. Two teaspoonfuls after meals. —*Ex.*

TREATMENT OF PIGMENTATION IN PREGNANT WOMEN.

The *Journal de Medicine de Paris* recommends the following to be rubbed in daily:

℞ Cocoa butter—castor oil, aa ij¼ grains.
Oxide of zinc, v grains.
Yellow oxide of mercury, ij grains
Essence of rose, q. s. —*Ev.*

EARACHE DROPS.

℞ Camphor-chloral m v.
Glycerine, m xxxiij.
Almond oil m xx.

M. Three drops of this mixture on absorbent cotton to be placed in the ear twice a day.

—*Chemist and Druggist.*

OINTMENT EOR THE ITCH.

R Creolini, grs. viiss.
Vaseline, oz. iss.

M. Sig. Anoint the affected p ırts once daily —Ex.

PEDICULUS PUBIS.

R Salicylic acid, 2 to 3 parts.
Toilet vinegar, 25 parts.
Alcohol, (85 per ct ), 75 parts.

M. Sig. The parts are to be rubbed with a piece of flannel wet with the mixture. In most cases a single ap. plication will be enough to destroy the pediculi.—Ex.

DEBILITY OF GRIPPE.

R Pepsine, scale, dr. iss.
Acid phosph. dil., dr. ij.
Syr. hypophosphites com, oz. ij.

Teaspoonful after meals in cold water. R, L. PATTERSON, M. D.
Bridgeville.

TONSILITIS.—Hudson:

R Tincture verat. vir. (Norwood) 30 drops.
Mo p. sulph., ½ grain.
Aq. dest., 6 drachms.

M. Sig. Teaspoonful every hour for two hours, and then every two or three hours, as needed.—Ex.

WHOOPING COUGH.

R Powd. belladonna root, 1-5 gr.
Dover's powder, ⅙ gr.
Sublimed sulphur, 4 grs.
White sugar, 10 grs.

M. Sig. Take in one dose from two to ten times a day, according to age of patient a d effect produced.—Ex.

AID TO DIGESTION.

R Ac. nitromuriat dil., jii drachms
Pepsin sacch., vj drachms.
Bismuth s. n. jv drachms.
Fl. ext. nucis vom., j drachms.
Hydrastis, iij drachms.
Glycerine, vj ounces.
Aq. vjii ounces.

Two drachms with meals.—Ex.

ECZEMA.

R Zinci oxidi, 1 ouuce.
Glycerine, 2 ounces.
Mucilag. Acaciæ, 2 ounces.

M. Sig. In extensive patches of eczema this paste is very agreeable. If itching is very severe, one per cent of carbolic acid may be added.

